# Individual and facility-level factors associated with women’s receipt of immediate postpartum family planning counseling in Ethiopia: results from national surveys of women and health facilities

**DOI:** 10.1186/s12884-021-04278-3

**Published:** 2021-12-05

**Authors:** Alexandria K. Mickler, Celia Karp, Saifuddin Ahmed, Mahari Yihdego, Assefa Seme, Solomon Shiferaw, Linnea Zimmerman

**Affiliations:** 1 Department of Population, Family and Reproductive Health, Johns Hopkins Bloomberg School of Public Health, 615 N. Wolfe St, Baltimore, MD USA; 2 School of Public Health, Addis Ababa University, Addis Ababa, Ethiopia

**Keywords:** Immediate postpartum family planning, Contraceptive counseling, Ethiopia, Maternal health, Reproductive health

## Abstract

**Background:**

Immediate postpartum family planning (IPPFP) helps prevent unintended and closely spaced pregnancies. Despite Ethiopia’s rising facility-based delivery rate and supportive IPPFP policies, the prevalence of postpartum contraceptive use remains low, with little known about disparities in access to IPPFP counseling. We sought to understand if women’s receipt of IPPFP counseling varied by individual and facility characteristics.

**Methods:**

We used weighted linked household and facility data from the national Performance Monitoring for Action Ethiopia (PMA-Ethiopia) study. Altogether, 936 women 5–9 weeks postpartum who delivered at a government facility were matched to the nearest facility offering labor and delivery care, corresponding to the facility type in which each woman reported delivering (*n* = 224 facilities). We explored women’s receipt of IPPFP counseling and individual and facility-level characteristics utilizing descriptive statistics. The relationship between women’s receipt of IPPFP counseling and individual and facility factors were assessed through multivariate, multilevel models.

**Results:**

Approximately one-quarter of postpartum women received IPPFP counseling (27%) and most women delivered government health centers (59%). Nearly all facilities provided IPPFP services (94%); most had short- and long-acting methods available (71 and 87%, respectively) and no recent stockouts (60%). Multivariate analyses revealed significant disparities in IPPFP counseling with lower odds of counseling among primiparous women, those who delivered vaginally, and women who did not receive delivery care from a doctor or health officer (all *p* < 0.05). Having never used contraception was marginally associated with lower odds of receiving IPPFP counseling (*p* < 0.10). IPPFP counseling did not differ by age, residence, method availability, or facility type, after adjusting for other individual and facility factors.

**Conclusion:**

Despite relatively widespread availability of IPPFP services in Ethiopia, receipt of IPPFP counseling remains low. Our results highlight important gaps in IPPFP care, particularly among first-time mothers, women who have never used contraception, women who delivered vaginally, and those who did not receive delivery care from a doctor or health officer. As facility births continue to rise in Ethiopia, health systems and providers must ensure that equitable, high-quality IPPFP services are offered to all women.

**Supplementary Information:**

The online version contains supplementary material available at 10.1186/s12884-021-04278-3.

## Background

Unintended and closely spaced births are associated with increased risks of maternal, neonatal, and child morbidity and mortality [1, 2]. The World Health Organization (WHO) recommends women and couples wait at least 24 months following childbirth before attempting the next pregnancy [3], yet estimates suggest that more than 61% of women in low- and middle-income countries (LMICs) have an unmet need for contraception in the postpartum period [4, 5]. Contraceptive counseling about postpartum family planning (PPFP) during antenatal (ANC) and postnatal care (PNC) can improve the use of contraception among women who wish to prevent pregnancy, thereby helping women achieve their reproductive goals.

Research on PPFP has historically focused on quantifying and understanding who initiates contraception in the first 12 months following childbirth [6]. Such studies have assessed the impact of integrating family planning services into ANC and/or PNC on women’s contraceptive intentions, use, and continuation, bolstering the evidence of the benefits contraceptive counseling throughout the continuum of care provides [7–13]. Facility-based childbirth also provides a unique window of opportunity for providers to discuss postpartum contraceptive options, as many family planning methods can be initiated soon after delivery [14–16]. Research from a number of LMICs suggests that offering immediate postpartum family planning (IPPFP) counseling and method provision as part of childbirth care can increase postpartum contraceptive use among women wishing to delay or limit pregnancy [10, 16–19].

To date, the majority of evidence on IPPFP counseling uses quasi-experimental designs to assess how counseling interventions impact postpartum women’s contraceptive use [7, 9, 10, 20–22]. For example, a study in Nepal indicated that women who received contraceptive counseling before discharge were significantly less likely to have an unmet need for family planning in the postpartum period; however, women’s overall receipt of IPPFP counseling was low [20]. Similarly, the integration of family planning counseling with delivery care was found to have a significant and positive association with postpartum contraceptive use in India, yet only 21.9% of women received IPPFP as part of delivery care [9]. Lacking in the current evidence base is a focus on the prevalence of, and factors contributing to, the provision of IPPFP counseling, including who receives counseling.

Some research suggests that the provision of IPPFP services is challenged by multiple facility-level factors, including a lack of skilled providers, disjointed maternal and family planning services, provider biases, and competing demands on the health system [9, 10, 23, 24]. Further, the decision of whether to initiate contraception immediately postpartum can be influenced by a variety of individual-level factors, including cultural or religious beliefs, partner disapproval, or concerns about method-related side-effects; such beliefs may also shape providers’ behavior, including if and who they counsel on postpartum methods [18, 25]. These factors mirror similar barriers faced by non-postpartum women and highlight the importance of family planning counseling that aligns with women and couple’s values and preferences [22, 26–28].

Recent data demonstrates that Ethiopia has made remarkable progress in improving facility-based delivery. Nearly half (48%) of all births in the past 5 years occurred in a health facility; this proportion has increased almost five-fold over the past decade from 10% in 2011 [29, 30]. In 2011, the Ethiopian Federal Ministry of Health (FMOH) integrated mandatory family planning counseling into all skilled maternity services, including immediate postpartum care, and outlines that “all health workers providing family planning services should have contraceptive clinical and counseling skills” [14]. Despite efforts to increase PPFP access and use, only 37.3% of postpartum women who delivered in a facility are estimated to use contraception by 12 months postpartum, signaling potential discrepancies between FMOH guidance and the postpartum care women receive [31]. Studies investigating PPFP in Ethiopia indicate greater PPFP use among women who delivered in a facility or in the presence of a skilled birth attendant [31, 32], but little is known about the factors associated with postpartum women’s receipt of IPPFP counseling.

Prior research on family planning service delivery suggests that facility quality metrics, such as the number of available family planning methods, and structural factors, including distance to the nearest facility, may impact contraceptive method choice or uptake among women of reproductive age [33–35]. Among married, non-postpartum women in rural Ethiopia, those who previously visited a facility, lived near a facility, or visited a facility with multiple methods in-stock were more likely to use contraception [36]. Similarly, women who live farther from a facility were less likely to use short-acting contraception [34]. However, most facility-based research focuses on all women of reproductive age, with limited exploration of how facility-level factors may impact postpartum women’s receipt of IPPFP services, specifically. Although the Demographic and Health Survey (DHS) and Service Provision Assessment (SPA) provide insight into individual and facility-based characteristics, respectively, sampling approaches generate temporally unique and spatially disjointed samples [34, 35]. This lack of linked and nationally representative data on postpartum women and their nearby facilities has resulted in a limited understanding of how individual characteristics and nearby service delivery environments relate to women’s receipt of IPPFP counseling. To our knowledge, no studies have assessed the relationships between postpartum women’s characteristics, IPPFP service availability, and whether women receive IPPFP counseling in Ethiopia.

This study aims to examine how women’s receipt of IPPFP counseling varies according to women’s characteristics and the contexts in which they receive labor and delivery care. Utilizing a national sample of recently postpartum women linked to nearby health facilities, our objectives were to 1) assess the proportion of postpartum women in Ethiopia who receive IPPFP counseling; and 2) identify individual and facility-level factors associated with the receipt of IPPFP counseling.

## Methods

### Conceptual framework

**Fig. 1:**
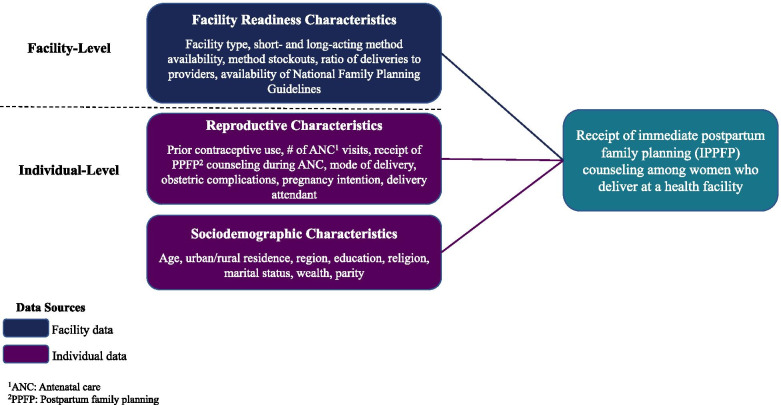
Conceptual framework informing study of immediate postpartum family planning counseling in Ethiopia

Our analysis was guided by a conceptual framework based informed by the literature. Figure 1 depicts factors hypothesized to influence women’s receipt of IPPFP counseling. The first level identifies women’s individual sociodemographic characteristics, such as age, parity, and residence. The second level includes women’s individual reproductive characteristics; for example, prior use of contraception, receipt of ANC, and mode of delivery. The second level addresses characteristics reflecting IPPFP service availability at the facility-level; for example, facility type, the monthly ratio of deliveries to available providers, and the availability of contraceptive methods. Drawing upon the literature, we hypothesize that these individual and facility factors individually and collectively inform whether women receive IPPFP counseling.

### Data

This cross-sectional analysis uses data from PMA-Ethiopia, a collaborative study between Addis Ababa University (AAU), the Ethiopian FMOH, and Johns Hopkins Bloomberg School of Public Health (JHSPH). PMA-Ethiopia generates timely cross-sectional and longitudinal data on reproductive, maternal, and newborn health indicators to inform national and regional government priorities and policies. PMA-Ethiopia’s survey follows pregnant or recently postpartum women through 1 year postpartum and is conducted in six regions, collectively representing 90% of Ethiopia’s population [37]. PMA-Ethiopia conducted a full census in 217 enumeration areas (EA) across six regions and listed 36,614 households between October and November 2019. All women aged 15–49 in these households were screened for participation in the survey and were eligible if they were pregnant during the survey or delivered within 9 weeks preceding the survey.

The baseline interview, conducted by resident enumerators, collected information about women’s sociodemographic characteristics, receipt of ANC, prior use of family planning, pregnancy intentions, and gestational age. Information about labor and delivery was collected at the follow-up interview; interview questions asked about pregnancy complications, delivery experiences, and postpartum experiences and behaviors, including whether women received IPPFP services. Women who were already 5–9 weeks postpartum at enrollment were asked the labor and delivery questions during the baseline interview. Informed consent to participate was obtained at the screening and prior to enrollment by all participants and was provided orally. Altogether, 2860 pregnant or recently postpartum women were enrolled in the survey. Women enrolled during pregnancy completed a baseline interview and a follow-up interview 6 weeks postpartum, with a follow-up rate of 88.9% (*n* = 2537) [37].

PMA-Ethiopia also conducts annual cross-sectional assessments of public and private facilities located in EAs used for the household survey. The Service Delivery Point (SDP) survey, herein referred to as the facility survey, collected data between September and November 2019 on facility characteristics and quality of care indicators for reproductive, maternal, and newborn health services, including family planning.

Our analysis uses 1) individual-level data collected from postpartum women who provided information about their delivery and postpartum experiences at the baseline or follow-up interviews; and 2) service availability data from the facility survey. All PMA-Ethiopia surveys received ethical approval from both the JHSPH [00009391] and AAU Institutional Review Boards [075/13/SPH] [37]. Additional information on the PMA Ethiopia survey design, including weighting and informed consent procedures, is described elsewhere [37, 38].

### Analytic sample

**Fig. 2:**
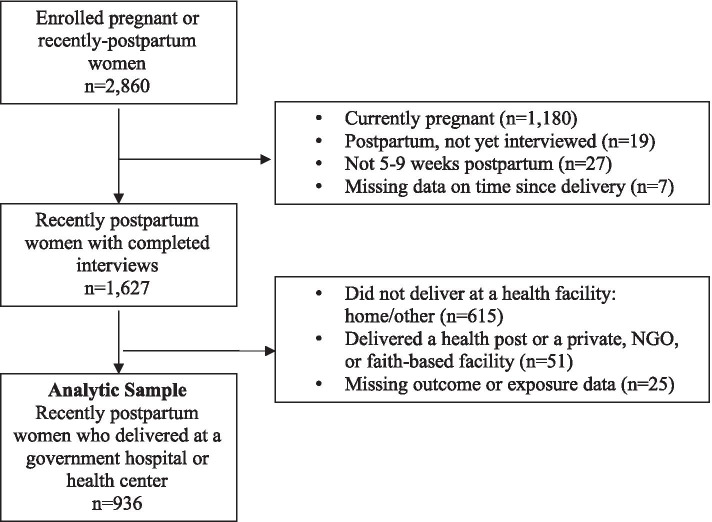
Analytic sample – postpartum women

Figure 2 presents the selection process for the analytic sample of postpartum women. Among the 2860 pregnant or recently postpartum women in our survey, we excluded pregnant women who had not yet delivered (*n* = 1180), those who had delivered but had not yet completed their follow-up interview (*n* = 19), those who were not within 5–9 weeks postpartum (*n* = 27), and women missing data on time since delivery (*n* = 7). Among the remaining 1627 eligible women with completed interviews, we excluded women who delivered at home, at a health post, or at other facilities unequipped to offer IPPFP services (*n* = 615), as information about IPPFP counseling and services was measured exclusively among women who delivered at a facility where family planning services could be provided immediately postpartum (hospital, health center). Due to the small number of women who delivered at private, faith-based, or non-governmental health facilities (only 3% of eligible women), we further restricted our sample to exclude women who did not deliver at a government facility (*n* = 51) and those with incomplete data on outcomes and exposures (*n* = 25). Our final analytic sample included 936 postpartum women who delivered in a government facility.

As we did not collect the name of the facility where each woman delivered, we matched each woman with the nearest facility that matched the facility type each woman reported attending for labor and delivery care. For example, if a woman reported delivering at a health center, we identified the nearest health center based on straight-line geodetic distance. Due to a lack of complete information on road networks, the point distance between each woman’s household and the nearest matching facility was used as a proxy for physical access. We identified 224 facilities as the closest matching facility type to women in our analytic sample; the characteristics of these facilities were utilized in our analysis. Supplemental Fig. S1 presents further information about the facility analytic sample selection.

### Measures

The primary dependent variable, receipt of IPPFP counseling, was assessed by asking women “*Before you left the facility, did a provider talk to you about a family planning method?*” We also assessed the proportion of women who received both IPPFP counseling and a contraceptive method, which was ascertained by asking women if they received a method before they left the facility, and if so, which method.

To assess individual-level correlates of IPPFP counseling, we measured a number of sociodemographic and reproductive health covariates from the household survey [37]. Sociodemographic variables included age, residence (urban/rural), region, education, religion, marital status, wealth, and parity. Reproductive health variables included number of ANC visits, receipt of PPFP counseling during ANC, mode of delivery (vaginal/Cesarean), prior use of contraception, pregnancy intention, type of delivery attendant, and experience of obstetric complications. We hypothesized that women who experienced more complicated deliveries because of obstetric complications would be less likely to receive IPPFP counseling due to the emergent nature of the delivery and competing healthcare priorities. Obstetric complications measured were self-reported severe bleeding, membrane leak or rupture without labor pain (> 24 h), membrane leak or rupture (at < 9 months gestation), malpresentation or malposition, prolonged labor (> 12 h), and convulsions or fits. These were assessed as any complications vs. no complications.

Facility-level characteristics included availability of IPPFP services, ascertained by asking the facility in-charge “*Is immediate postpartum family planning provided at this facility?*”. Additional facility characteristics included short- and long-acting method availability, recent stockouts, ratio of monthly deliveries to providers, and presence of family planning guidelines. We defined method availability by whether each of five non-barrier family planning methods appropriate for IPPFP were in-stock and observed on the day of the survey. These included long-acting methods; specifically, implants and non-hormonal intrauterine devices (IUDs), and the following short-acting methods: progesterone-based pills, progestin-based injectables, and emergency contraception [14, 39]. The proportion of women who reported using exclusive breastfeeding as their primary contraceptive method was calculated among women who answered “*No*” to the question, *“Do you plan to use a method of family planning, other than breastfeeding, within a year of giving birth*?” Recent method stockout was defined by whether any method was reported out-of-stock at any time in the past 3 months, and if so, which method (short-acting, long-acting, or both). We also examined the ratio of monthly deliveries to providers, as a proxy for caseload volume, as a categorical variable and generated tertiles based on facility distributions. Finally, we explored the presence of national family planning guidelines in the delivery room of each facility.

### Analyses

We used descriptive statistics to examine the distribution of women’s sociodemographic and reproductive characteristics (Table 1) and receipt of IPPFP methods, among women who received IPPFP counseling (Table 2). We described facility characteristics in two ways; first by the distribution of characteristics of the 224 linked facilities and second, by the percentage of women who delivered in a facility of each type (Table 3). We assessed the bivariate distributions of IPPFP counseling and each covariate at both levels (Individual, Table 1; Facility, Table 3). We used multivariate, multilevel models to estimate differences in women’s odds of receiving IPPFP counseling by individual and facility-level characteristics, adjusting for clustering of women within the facilities. Our final adjusted model included individual and facility-level covariates driven by theory and conceptual relevance, while also accounting for established confounders of women’s receipt of reproductive health services (i.e. age and urban/rural residence) (Table 4) [40, 41]. We examined model fit by analyzing model fit statistics (i.e., Aikake’s Information Criterion (AIC) values) and assessed collinearity at 0.6 using a correlation matrix for all analytic variables. We excluded wealth from the multivariate analysis due to its high correlation with urban/rural residence (*r* = 0.749) and religion due to small cell sizes, limiting statistical power. Statistical significance for the adjusted multivariate analysis was set to *p* < 0.05. All analyses were weighted to reflect the national population of pregnant and postpartum women in Ethiopia and accounted for the complex survey design [42]. All analyses were conducted in StataSE, Version 16 [43].

**Table 1:** Sociodemographic and reproductive characteristics of postpartum Ethiopian women who delivered at a government health facility (*n* = 936)^b^

	Total		Received IPPFP counseling		Did not receive IPPFP counseling		***P***-value
	N	% (col)	N	% (col)	N	% (col)	
**Total**	936	100.0	251	26.8	685	73.2	
**Sociodemographic characteristics**							
**Age**							
15–19	112	12.0	22	8.8	90	13.2	0.326
20–34	686	73.2	194	77.3	492	71.7	
35+	138	14.8	35	13.9	103	15.1	
**Urban/Rural residence**							
Rural	583	62.3	156	62.0	427	62.4	0.940
Urban	353	37.7	95	38.0	257	37.6	
**Region**							
Addis	53	5.7	20	7.9	34	4.9	0.368
Afar	5	0.6	1	0.2	5	0.7	
Amhara	223	23.8	50	19.9	173	25.2	
Oromia	377	40.2	103	41.2	273	39.9	
Southern Nations, Nationalities, and Peoples’ Region	185	19.8	48	19.2	137	20.0	
Tigray	93	9.9	29	11.7	63	9.2	
**Education**							
Never attended	264	28.2	71	28.3	193	28.2	0.513
Primary	418	44.6	120	47.7	298	43.5	
Secondary or Higher	254	27.2	60	24.0	193	28.3	
**Religion**							
Protestant	214	22.9	61	24.2	154	22.4	0.028
Orthodox	424	45.3	130	51.9	293	42.9	
Muslim	288	30.7	54	21.5	264	34.1	
Catholic/Wakefeta/Other	10	1.1	6	2.3	4	0.6	
**Marital status**							
Never married/widowed/separated	20	2.1	6	2.6	13	1.9	0.550
Married/Living with partner	916	97.9	245	97.4	671	98.1	
**Wealth quintile**							
Lowest	96	10.3	20	8.0	76	11.1	0.495
Lower	139	14.8	41	16.2	98	14.3	
Middle	169	18.1	54	21.6	114	16.7	
Higher	226	24.1	53	21.3	172	25.2	
Highest	306	32.7	83	32.9	224	32.7	
**Parity**							
1	327	34.9	61	24.2	266	38.8	< 0.001
2–3	345	36.9	118	47.1	227	33.2	
4+	264	28.2	72	28.7	192	28.0	
**Reproductive characteristics**							
**Number of ANC visits**							
0	118	12.4	31	12.4	85	12.4	0.447
1–3	391	41.9	95	37.9	296	43.4	
4+	427	45.7	125	49.8	302	44.2	
**Received PPFP counseling during ANC**^**a**^							
No	711	86.9	161	74.0	549	91.6	< 0.001
Yes	107	13.1	57	26.0	50	8.4	
**Delivery mode**							
Vaginal	840	89.8	206	81.9	635	92.6	< 0.001
Cesarean Section	96	10.2	45	18.1	50	7.4	
**Obstetric complications**							
None	501	53.5	132	52.5	370	53.9	0.765
Any	435	46.5	119	47.5	315	46.1	
**Prior use of contraception**							
No	291	31.1	59	23.3	232	33.9	0.011
Yes	645	68.9	192	76.7	453	66.1	
**Pregnancy intention**							
Wanted	628	67.1	169	67.3	459	67.0	0.942
Unintended	308	32.9	82	32.7	226	33.0	
**Delivery attendant**							
Skilled Attendant/Other	348	37.2	73	29.0	275	40.2	0.008
Nurse/Midwife	419	44.7	62	24.8	107	15.6	
Doctor/Health Officer	169	18.1	116	46.2	302	44.2	
**Distance to closest facility (km), mean (SE)**	5.96 (0.48)		6.47 (0.73)		5.77 (0.53)		

^a^Among women who received at least 1 ANC visit

^b^All values were weighted to account for the complex survey design

**Table 2:** Receipt of IPPFP methods or referrals among women who received IPPFP counseling (*n* = 251^a^)

	N^**a**^	%^**b**^
**No method or referral**	206	79.8
Report using exclusive breastfeeding as family planning^c^	36	17.8
**Received a referral**	**17**	**6.6**
**Received a contraceptive method**	**35**	**13.6**
Method Received		
Implant	30	87.2
Injectables	4	11.9
Female Sterilization	1	1.9
Contraceptive pills	0	0.0
Non-hormonal IUD	0	0.0
Emergency contraception	0	0.0

^a^Unweighted

^b^Weighted

^c^Among women who received IPPFP counseling, but did not receive a method or a referral

**Table 3:** Facility-level (*n* = 224) and woman-level (*n* = 936) descriptive characteristics of linked health facilities

**Facility-level, linked facility characteristics (*n*** **= 224)**	**Total (*n*** **= 224)**	**Health Centers (*n*** **= 136)**	**Hospitals (*n*** **= 88)**	*p*-value*		
	**%**	**%**	**%**			
	100	60.7	39.3			
**Provides IPPFP services**				0.001		
Yes	93.7	89.7	100			
**Availability of long-acting, reversible (LARC) methods**				0.002		
0–1 LARC method	13.4	19.1	4.6			
2 LARC methods	86.6	80.9	95.5			
**Availability of 3 short-acting (SA) methods**				0.359		
Yes	71.4	73.5	72.7			
**Method stockouts in the last 3 months**				0.226		
No stockouts	60.3	55.9	67.1			
Either LARC or SA stockout	31.7	35.3	26.1			
Both LARC & SA stockout	8	8.8	6.8			
**Ratio of deliveries to providers (monthly)**				< 0.001		
Low	33.5	27.9	42.1			
Medium	33.5	26.5	44.3			
High	33	45.6	13.6			
**National Family Planning Guidelines available on-site**				0.030		
Yes	51.8	48.8	56.8			
**Woman-level, linked facility characteristics (*n*** **= 936)**	**Total (*n*** **= 936)**	**Health Centers (*n*** **= 548)**	**Hospitals (*n*** **= 388)**	**Received IPPFP Counseling (*n*** **= 251)**	**Did not receive IPPFP counseling (*n*** **= 685)**	*p*-value**
	**%**	**%**	**%**	**%**	**%**	
	100	58.6	41.4	26.8	73.2	
**Provides IPPFP services**						0.327
Yes	92.5	87.3	100	95.0	8.4	
**Availability of long-acting, reversible (LARC) methods**						
0–1 LARC method	10.6	16.6	2.2	10.8	10.6	
2 LARC methods	89.4	83.4	97.8	89.2	89.4	
**Availability of 3 short-acting (SA) methods**						0.058
Yes	68.8	65.9	72.9	61.9	38.1	
**Method stockouts in the last 3 months**						0.744
No stockouts	58.8	55.2	64	57.6	89.3	
Either LARC or SA stockout	33.5	37.3	28.2	35.7	32.7	
Both LARC & SA stockout	7.7	7.5	7.8	6.7	8.0	
**Ratio of deliveries to providers (monthly)**						0.945
Low	20.4	14.3	29.1	21.2	20.2	
Medium	33.2	23.5	46.9	33.6	33.0	
High	46.4	62.2	24	45.2	46.8	
**National Family Planning Guidelines available on-site**						0.706
Yes	50.1	46.1	55.8	48.5	50.7	

*LARC* Long-acting and reversible contraception, *SA* Short-acting contraception

*Represents the design-based chi-squared test between each facility-level characteristic and government facility type (health center/hospital)

**Represents the weighted bivariate logistic regression between each woman-level facility characteristic and women’s receipt of IPPFP counseling

**Table 4:** Adjusted odds of receiving immediate postpartum family planning counseling by women’s individual and facility-level characteristics (*n* = 936)

	AOR	95% CI		***P***-value
**Individual-Level (Sociodemographic and Reproductive) Characteristics**				
**Age**				
15–19	*ref*			
20–34	1.12	0.51–2.42		0.780
35+	0.91	0.36–2.33		0.846
**Urban/Rural**				
Rural	*ref*			
Urban	0.87	0.49–1.55		0.630
**Parity**				
1	*ref*			
2–3	2.54**	1.46–4.39		0.001
4+	2.20**	1.06–4.56		0.035
**Prior use of contraception**				
No	*ref*			
Yes	1.62*	0.97–2.70		0.064
**Delivery mode**				
Vaginal	*ref*			
Cesarean Section	3.39**	1.68–6.81		0.001
**Delivery attendant**				
Skilled Attendant/Other	*ref*			
Doctor/Health Officer	2.30**	1.24–4.28		0.009
Nurse/Midwife	1.50	0.83–2.71		0.175
**Facility-Level Characteristics**				
**Facility Type**				
Government Health Center	*ref*			
Government Hospital	0.83	0.45–1.55		0.561
**Availability of long-acting, reversible methods**				
0–1 LARC method	*ref*			
2 LARC methods	0.97	0.41–2.28		0.942
**Availability of all 3 short-acting methods**				
No	*ref*			
Yes	0.69	0.38–1.23		0.204

*AOR* Adjusted Odds Ratio, *LARC* Long-acting and reversible contraception, *SA* Short-acting contraception

*p < 0.10, **p < 0.05

## Results

### Individual characteristics

Table 1 summarizes the sociodemographic and reproductive characteristics of our sample. On average, women were 26.5 years old and the majority lived in rural areas (62.3%). The largest proportion of women lived in the Oromiya region (40.2%) and most completed primary education or lower (72.8%). The sample was predominately Orthodox (45.3%) and nearly all women were married (97.9%). About half of the sample was of the “higher” or “highest” wealth quintiles (56.8%). Over one-third of women (34.9%) were primiparous after delivery.

While most women had at least one ANC visit (87.6%), fewer than half completed four or more visits. Among women who reported at least one ANC visit, only 13.1% received counseling on PPFP during ANC. Ten percent of women delivered via Caesarean and nearly half of all women (46.5%) self-reported experiencing at least one obstetric complication. The majority of women had previously used family planning (68.9%) and reported that their most recent pregnancy was wanted when they became pregnant (67.1%). Forty-five percent were delivered by a nurse or midwife whereas 18.1% were attended by a doctor or health officer. On average, women lived 5.96 km (SE = 0.48) from their linked health facility.

### Receipt of IPPFP counseling and family planning method initiation

Roughly one-quarter (26.8%) of women who delivered in a health facility received IPPFP counseling (Table 1). Among women who received IPPFP counseling, most women (79.8%) did not receive a method or a referral; only 13.6 and 6.6% of women received a contraceptive method or a referral, respectively, immediately postpartum (Table 2). Among women who received IPPFP counseling and a contraceptive method, implants were the most common (87.2%), followed by progestin-only injectables (11.9%) and sterilization (1.6%). No women reported initiating non-hormonal IUDs, receiving hormonal birth control pills, or receiving emergency contraceptive pills immediately postpartum. Among women who received IPPFP counseling but did not receive a family planning method or referral, 17.8% reported using exclusive breastfeeding as a family planning method.

### Service delivery environment

The first half of Table 3 presents the characteristics of the 224 linked government facilities included in our sample; the second half presents the distribution of these facility characteristics at the woman-level, reflecting the proportion of woman whose linked facility reported each characteristic included in our analysis.

Most linked facilities reported providing IPPFP services (93.7%) and had both long-acting methods in-stock (86.6%). Nearly three-quarters of facilities had all three short-acting methods in-stock (71.4%). Although more than half (60.3%) of facilities reported no method stockouts in the past 3 months, approximately one-third (31.7%) had either a long-acting or short-acting method stockout and 8.0% had stockout of both method types. Almost half (45.6%) of health centers had a high monthly ratio of deliveries to provider, whereas the majority of hospitals reported a low or medium ratio of deliveries per provider (86.4%). About half (51.8%) of all facilities had the Ethiopian National Family Planning Guidelines available on-site.

More than half of women delivered at a government health center (58.6%), while the remaining 41.4% of women delivered at a government hospital. At the individual-level, almost all women were linked to a facility that provided IPPFP services (92.5%) and had both long-acting methods in-stock (89.4%). Sixty-nine percent of women were linked to a facility with all short-acting methods in-stock. Over half of women (58.8%) were matched to facilities with no recent method stockouts, with one-third linked to facilities with either long-acting or short-acting recent method stockouts. Among women who delivered at a health center, most were matched to a health center with a high ratio of monthly deliveries per provider (62.2%), whereas almost half of women who delivered at a hospital were linked to a facility with a medium ratio of deliveries per provider (46.9%).

### Factors associated with IPPFP counseling

Results from the multivariate, multilevel model, which adjusted for age, residence, parity, prior use of contraception, delivery type, delivery attendant, facility type, and contraceptive method stocks, are presented in Table 4. Women with two or three children (AOR: 2.54, CI: 1.46–4.39) and those with more than four children (AOR: 2.20, CI: 1.06–4.56), women who delivered via Cesarean section (AOR: 3.39, CI: 1.68–6.81), and women who delivered in the presence of a doctor or health officer (AOR: 2.30, CI: 1.24–4.28) were significantly more likely to receive IPPFP counseling**.** Women who ever previously used contraception had marginally higher odds of receiving IPPFP counseling (AOR: 1.62, CI: 0.97–2.70); (*p* < 0.10). We observed no significant differences in receipt of IPPFP counseling by age, residence, or any facility-level characteristics.

## Discussion

Our study, conducted among a national sample of recently postpartum women and the government facilities in which they delivered in Ethiopia, found that despite high availability of IPPFP counseling and services among facilities, only one in four women were counseled about contraception immediately after delivery. Receipt of IPPFP counseling varied according to women’s sociodemographic and reproductive characteristics, including how many children they had, their prior use of contraception, and their delivery experiences; specifically, Cesarean section delivery and provider type. No facility characteristics or factors reflecting IPPFP service readiness were associated with women’s receipt of IPPFP counseling.

Women with more than one child, including their most recent birth, were significantly more likely to receive IPPFP counseling than women who had just delivered their first child. This may reflect potential provider biases against offering family planning to first-time mothers, given cultural beliefs or social norms about childbearing or feared health consequences of contraception [44–46]. Contraceptive non-use has been linked to individual, community, and provider-level concerns that modern contraceptives may cause infertility, particularly among young, nulliparous women [45, 47]. This belief can be particularly prevalent in regions where a larger family size is associated with perceived community, religious, or intra-familial respect [47, 48]. Alternatively, this finding could suggest that multiparous women are more likely to receive IPPFP counseling due to their familiarity with the health system, or could indicate targeted counseling by providers to a population of women who providers’ perceive have already achieved or are close to meeting their reproductive goals. Nationally representative and region-specific data suggest that PPFP use is significantly higher among Ethiopian women with fewer children and that PPFP uptake can drop by half, or is adopted much later in the postpartum period, among women with four or more births [17, 49]. Despite these trends, FMOH and WHO guidelines state that contraceptive counseling and services should be offered to *all* postpartum women, regardless of parity [3, 14]. Our results signal the need for more equitable IPPFP service provision and targeted efforts to ensure primiparous women are counseled accordingly.

We anticipated that women experiencing more complicated deliveries would be less likely to receive IPPFP counseling due to the emergent nature of the delivery and competing healthcare priorities. While we did not observe an association between obstetric complications and receipt of IPPFP counseling, we found that women who delivered via Cesarean section (approximately 10% of our sample) were significantly more likely to be counseled on postpartum contraception than those who delivered vaginally. Estimates suggest that between 2 and 18% of all facility-based deliveries in Ethiopia occur via Cesarean section; such deliveries are often initiated due to or may result in adverse maternal or neonatal outcomes [29, 50–52]. Consequently, women who deliver via Cesarean section may have existing complications or be at heighted risk for future health issues, thereby increasing the need for birth spacing or limiting. Alternatively, these findings may simply reflect increased opportunities for IPPFP counseling and service provision due to prolonged contact with the health system with obstetric surgery and time spent in the facility for recovery. Nonetheless, Ethiopia’s FMOH guidelines suggest that providers should ensure all women have access to IPPFP services, regardless of delivery mode.

We observed significant disparities in whether women were counseled on contraception immediately postpartum based on the type of provider who cared for them during labor and delivery. Ethiopia’s FMOH guidelines indicate that all healthcare workers providing family planning services should be equipped with contraceptive counseling skills, including doctors, nurses, health officers, and midwives [14]. While we were unable to evaluate the competency of providers to deliver IPPFP counseling, our findings underscore the need for further research on how provider practices, often rooted in both occupational and community-level norms, may influence women’s receipt of IPPFP counseling and services [44–47]. Such evidence would provide useful insights to improve postpartum women’s receipt of facility-based IPPFP services.

Nearly all women in our sample were linked to facilities with a high degree of IPPFP service availability, including a wide range of method availability, trained providers, and minimal method stockouts, yet we found that women were no more likely to have been counseled on IPPFP based upon these facility characteristics, reinforcing the notion that the availability of services and commodities alone does not translate to service provision. This finding echoes similar research regarding reported versus actual facility readiness to provide labor and delivery care among healthcare providers in Addis Ababa. Despite the development of referral networks to optimize use of healthcare resources and adequate staffing, qualitative data from maternal healthcare providers suggests that a lack of updated and consistent training prevents women from receiving basic emergency obstetric care [53]. Similarly, evidence from northwest Ethiopia indicates that while antenatal and delivery care was available in most facilities, many basic elements of care were provided inconsistently or incorrectly, indicating the need to improve the functional capacity of health providers [54]. Our findings similarly suggest that facility readiness to provide IPPFP services does not necessarily translate into women’s receipt of services.

Alternatively, this discordance between facility readiness and women’s receipt of IPPFP counseling may suggest differences in the government-recommended and provider-preferred timing for contraceptive counseling in Ethiopia. With competing demands for maternal and newborn health in the postpartum period, providers may prioritize other care and refrain from initiating discussions about contraception or refer women to lower-level facilities or community-based services to receive family planning. Recent research indicates that the integration of PPFP counseling and services into Ethiopia’s community-level health extension worker (HEW) services may compound the benefits of offering IPPFP at the facility-level [17, 55]. Evidence suggests that women who delivered in a facility and also received PPFP information and services from a HEW were more likely to adopt contraception in the postpartum period; perhaps highlighting the benefit of counseling at multiple times in the postpartum period and/or women’s preference to initiate contraception later after childbirth [55].

We found that women who ever used contraception had marginally higher odds of receiving IPPFP counseling, a finding which has been associated with PPFP use in multiple contexts, including Ethiopia [9, 56–59]. This association may suggest that due to their familiarity with contraceptive methods, prior family planning users are more likely to engage in a conversation about IPPFP [56, 57]. Conversely, this may also indicate a hesitancy among providers to counsel women who never used family planning to adopt a method for the first time immediately postpartum [44, 47]. However, the immediate postpartum period has been established as an opportune time to discuss or initiate contraception, particularly among new users and women in low-resourced settings; such women may experience limited opportunities to return to a facility for additional postnatal care [16, 23, 39, 60, 61]. To ensure all postpartum women have access to IPPFP counseling, information, and services, providers must ensure family planning discussions are not limited to women who already have experience with contraception.

Our study is not without limitations. Facilities surveyed as part of this study are not nationally representative, and instead reflect those serving areas where women surveyed live. Facility characteristics may not be generalizable across all regions of Ethiopia, nor do they reflect readiness to deliver key reproductive health services among all public facilities offering labor and delivery services. Our sample was restricted to public facility-based births; therefore, we did not assess the extent to which IPPFP counseling occurred at home or at private, NGO, or faith-based facilities. Receipt of IPPFP counseling may vary considerably by women’s characteristics (e.g., wealth) and service delivery environments in these contexts. Future research on the receipt and availability of IPPFP outside of public facilities is needed to understand the availability and receipt of IPPFP services among women who seek care in non-governmental facilities. Additionally, while the facility survey mainly collected information on supply-side factors relating to IPPFP service delivery, expanded measures on counseling quality, including content, knowledge retention, and documentation of informed non-use, could further identify disparities in women’s receipt of high-quality IPPFP counseling and services [19, 21, 62]. Lastly, we hypothesized that women delivered at their nearest facility offering labor and delivery services and matched women based on the type of facility they reported attending. We believe this to be a reasonable assumption given that the average distance to a health facility is over five kilometers and service density is low [34]; however it is possible that women delivered elsewhere, particularly given that facility choice can be driven by factors other than distance, such as perceptions regarding facility quality, wait time, or confidentiality [23, 63].

Nonetheless, our study has a number of strengths, most notably our ability to geographically link nearly nationally representative data collected from women with information from nearby facilities to understand factors associated with women’s receipt of IPPFP counseling. Importantly, we expand upon existing research, which often focuses on the impact individual or facility-level characteristics on postpartum contraceptive use alone, by examining how these factors individually and collectively influence postpartum women’s receipt of IPPFP counseling.

## Conclusion

Despite a relatively high degree of service availability and readiness, receipt of IPPFP counseling is low among women who deliver in public facilities in Ethiopia, and significant disparities in counseling exist. Concerted programmatic and clinical efforts must ensure that IPPFP counseling and services are equitably offered to all women, regardless of women’s delivery mode, parity, provider type, or experience with contraception. Future research should explore factors that could improve the equitable delivery of IPPFP counseling among postpartum women in Ethiopia.

## Supplementary Information

**Additional file 1: Supplemental Figure S1.** Analytic Sample –Health Facilities.

**Additional file 2: Supplemental Table 1.** Bivariate associations between facility-level characteristics and women’s receipt of IPPFP counseling.

## Data Availability

The datasets analysed in the current study are available in the Performance Monitoring for Action repository, https://www.pmadata.org/data/request-access-datasets.

## Abbreviations

AAU: Addis Ababa University
DHS: Demographic and Health Survey
EA: Enumeration area
FMOH: Federal Ministry of Health
HEW: Health extension worker
IPPFP: Immediate postpartum family planning
JHSPH: Johns Hopkins School of Public Health
PMA: Performance Monitoring for Action
PPFP: Postpartum family planning
SDP: Service Delivery Point
SPA: Service Provision Assessment
WHO: World Health Organization
