# Supplementary figures and images for: Individual and facility-level factors associated with women’s receipt of immediate postpartum family planning counseling in Ethiopia: results from national surveys of women and health facilities
Source: BMC Pregnancy Childbirth. 2021 Dec 5;21:809. doi: 10.1186/s12884-021-04278-3 (PMC8645155; doi:10.1186/s12884-021-04278-3)

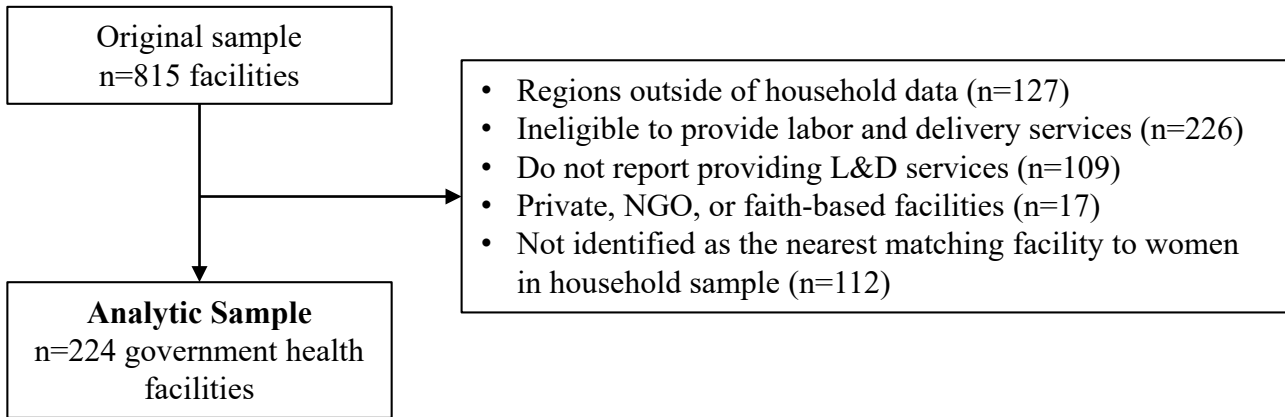

**Supplemental Figure S1: Analytic Sample – Health Facilities**

Supplement: Supplementary file 1 — Additional file 1: Supplemental Figure S1. Analytic Sample –Health Facilities. [file 12884_2021_4278_MOESM1_ESM.pdf]
