# Supplementary material for: Individual and facility-level factors associated with women’s receipt of immediate postpartum family planning counseling in Ethiopia: results from national surveys of women and health facilities
Source: BMC Pregnancy Childbirth. 2021 Dec 5;21:809. doi: 10.1186/s12884-021-04278-3 (PMC8645155; doi:10.1186/s12884-021-04278-3)
Supplement: Supplementary file 2 — Additional file 2: Supplemental Table 1. Bivariate associations between facility-level characteristics and women’s receipt of IPPFP counseling. [file 12884_2021_4278_MOESM2_ESM.docx]

**Supplemental Table 1: Bivariate associations between facility-level characteristics and women’s receipt of IPPFP counseling**

|  | **Receipt of IPPFP Counseling (%)** | | **p-value** |
| --- | --- | --- | --- |
|  | **Yes** | **No** |  |
| **Facility Type** | | | |
| Health Center | 26.3 | 73.7 | 0.763 |
| Hospital | 27.6 | 72.4 |  |
| **Provides IPPFP Services** |  |  |  |
| No | 17.9 | 82.1 | 0.327 |
| Yes | 27.5 | 72.4 |  |
| **Availability of long-acting, reversible methods** | | |  |
| 0-1 LARC | 10.6 | 89.4 | 0.935 |
| 2 LARCs | 10.8 | 89.2 |  |
| **Availability of 3 short-acting methods** | | |  |
| No | 32.8 | 67.2 | 0.058 |
| Yes | 24.2 | 75.8 |  |
| **Method stockouts in the last 3 months** | | |  |
| No stockouts | 26.3 | 73.7 | 0.744 |
| Either LARC or SA stockout | 28.6 | 71.4 |  |
| Both LARC & SA stockouts | 23.4 | 76.6 |  |
| **Ratio of deliveries to providers (monthly), mean (SD)** | | |  |
| Low | 28.4 | 71.6 | 0.931 |
| Medium | 26.4 | 73.6 |  |
| High | 26.5 | 73.5 |  |
| **National Family Planning Guidelines available on-site** | | |  |
| No | 27.7 | 72.3 | 0.706 |
| Yes | 26.0 | 74.0 |  |

LARC = Long-acting and reversible contraception

SA = Short-acting contraception
